# Experimental treatment of *NRAS*-mutated neurocutaneous melanocytosis with MEK162, a MEK-inhibitor

**DOI:** 10.1186/2051-5960-2-41

**Published:** 2014-04-08

**Authors:** Heidi VN Küsters-Vandevelde, Annelieke ECAB Willemsen, Patricia JTA Groenen, Benno Küsters, Martin Lammens, Pieter Wesseling, Melika Djafarihamedani, Jos Rijntjes, Hans Delye, Michel A Willemsen, Carla ML van Herpen, Willeke AM Blokx

**Affiliations:** 1 Department of Pathology, Canisius Wilhelmina Hospital, PO Box 9015, 6500 GS Nijmegen, The Netherlands; 2 Department of Medical Oncology, Radboud University Medical Center, PO Box 9100, 6500 HB Nijmegen, The Netherlands; 3 Department of Pathology, Radboud University Medical Center, PO Box 9100, 6500 HB Nijmegen, The Netherlands; 4 Department of Pediatric Neurology, Radboud University Medical Center, PO Box 9100, 6500 HB Nijmegen, The Netherlands; 5 Department of Neurosurgery, Radboud University Medical Center, PO Box 9100, 6500 HB Nijmegen, The Netherlands; 6 Department of Pathology, VU University Medical Center, PO Box 7057, 1007 MB Amsterdam, The Netherlands; 7 UZA, University of Antwerp, Wilrijkstraat 10, 2650 Edegem, Belgium

**Keywords:** NRAS, Neurocutaneous melanosis, Leptomeningeal melanocytosis, Targeted therapy, MEK inhibitor, MEK162

## Abstract

Neurocutaneous melanosis (NCM) is a rare congenital disorder characterized by the association of large and/or multiple congenital melanocytic nevi (CMN) of the skin with melanocytic lesions of the leptomeninges, including melanocytosis. Leptomeningeal melanocytosis carries a poor prognosis once neurological symptoms develop. Despite surgery, which is often not radical, few other treatment options exist. Recently, it was demonstrated that early embryonic, post-zygotic somatic mutations in the *NRAS* gene are implicated in the pathogenesis of NCM.

In this report, we present a 13-year-old boy with NCM and progressive symptomatic leptomeningeal melanocytosis. A somatic NRAS^Q61K^ mutation was present in both CMN as well as the melanocytosis. Despite repeated surgery, the patient showed clinical progression. Therefore, treatment with MEK162, a MEK inhibitor, was started on compassionate use base. The patient died only five days later, i.e. too early to expect a clinical effect of MEK162 therapy. We therefore studied the effect of MEK162 at the protein level in the leptomeningeal tumor by immunohistochemical and Western Blot analyses using Ki67 and pERK antibodies. We observed lower MIB-1 expression and lower pERK expression in the post-treatment samples compared to pre-treatment, suggesting a potential effect of MEK inhibiting therapy. Further studies are needed to determine whether MEK inhibitors can effectively target *NRAS*-mutated symptomatic NCM, a rare but potentially fatal disease.

## Background

Neurocutaneous melanosis (NCM) is a rare congenital disorder first described by Rokitansky in 1861, in which affected patients have an increased number of melanocytes in the leptomeninges of the central nervous system (CNS) and the skin [1,2]. The diagnostic criteria for NCM, originally proposed by Fox [3] and revised by Kadonaga and Frieden [1] include large or multiple congenital melanocytic nevi (CMN) of the skin associated with leptomeningeal melanocytosis or (primary) leptomeningeal melanoma. The pathogenesis of NCM has not been fully elucidated but it is thought to represent a congenital error during early embryonic migration of melanocytes.

Leptomeningeal melanocytosis consists of a diffuse proliferation of histologically benign appearing melanocytes in the leptomeninges, without evident invasion of the CNS [4]. Patients can develop neurological symptoms due to impaired CSF drainage resulting in increased intracranial pressure and/or due to mass effect on the brain or spinal cord. Even in the absence of malignant progression, leptomeningeal melanocytosis carries a poor prognosis once neurological symptoms develop [1,5].

So far, surgical resection of the leptomeningeal tumor is the treatment of choice, but complete resection is often impossible. In addition, the value of chemotherapy and radiotherapy is not clear [1]. Recently, a few studies have demonstrated that early embryonic, post-zygotic somatic mutations in the *NRAS* gene are implicated in the development of NCM [6,7].

In this case report, we present a 13-year-old boy with *NRAS*-mutated leptomeningeal melanocytosis in the context of NCM. Despite repeated surgery, the patient showed clinical progression and treatment with MEK162, a MEK inhibitor, was started on compassionate use. Unfortunately, the patient died only five days after start of this treatment, i.e. too early to expect a clear clinical effect. We therefore proceeded with investigating the potential effect of MEK162 at the protein level by immunohistochemical and Western Blot analyses using Ki67 and pERK antibodies. We observed lower MIB-1 expression and lower pERK expression in the post-treatment melanocytosis samples compared to the pre-treatment sample, suggesting a potential effect of MEK inhibiting therapy.

## Case presentation

### Patient history

**Figure 1:**
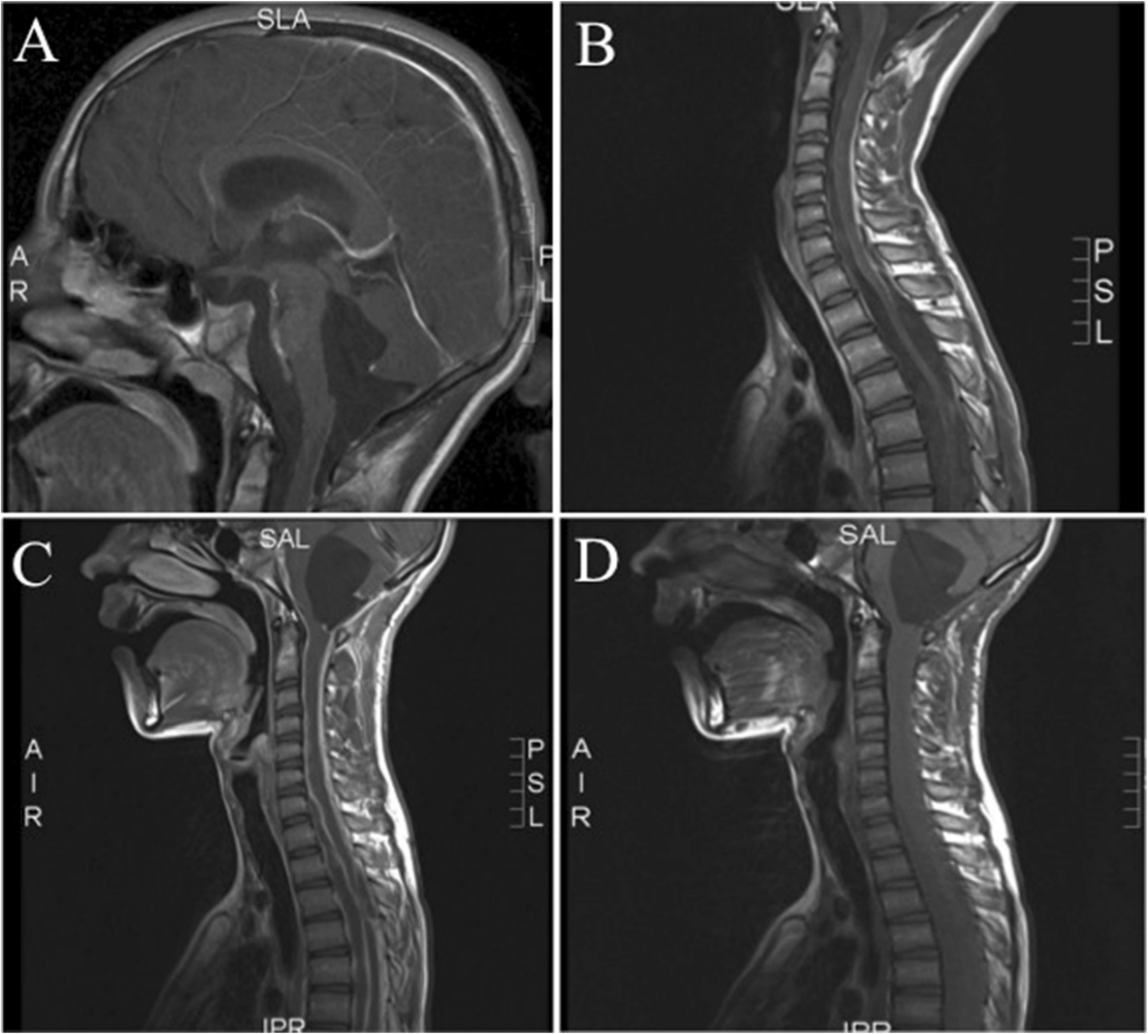
**Brain and spinal MRI images. A**. MRI of the brain showed an enlarged fourth ventricle in contact with a retrocerebellar cyst extending through the foramen magnum, and an enlarged subarachnoid cistern anterior to the brainstem, extending from the medulla oblongata towards the floor of the third ventricle. The brain stem appeared to be compressed or atrophied due to the combined pressure from these cysts. T1 weighted image with contrast. **B**. Spinal MRI showed a widened spinal canal and a distorted and atrophic spinal cord, due to the presence of multiple cysts with extensive leptomeningeal and dural contrast enhancement without the presence of solid masses. T1 weighted image with contrast. **C** and **D**. MRI of the brain and spinal cord, a few months after surgery, showed progressive leptomeningeal lesions of the brain and spinal cord with (further) compression of these structures and hydrocephalus (**C** with, **D** without contrast).

A 13-year-old boy was admitted to the outpatient clinic of pediatric neurology with progressive neurological symptoms. Since birth, he had multiple large melanocytic nevi of the skin (trunk, legs, arms, face) for which multiple resections were performed in the past. As a child, his developmental milestones for speech and fine motor skills were slightly delayed. Now, he presented with vertigo, headache, nausea, vomiting and pain in his legs. Neurological examination revealed bilateral papilloedema, slurred speech, dysmetria and dysdiadochokinesis of both arms, and an unsteady gait due to cerebellar ataxia plus mild pyramidal tract and sensory involvement. Knee and ankle jerk reflexes were clearly increased, but plantar responses were normal. MRI of the brain showed enhanced signal intensity of the uncus, an enlarged fourth ventricle in contact with a retrocerebellar cyst extending through the foramen magnum, and an enlarged subarachnoid cistern anterior to the brainstem, extending from the medulla oblongata towards the floor of the third ventricle. The brain stem appeared to be compressed or atrophied due to the combined pressure from these cysts (Figure 1A). Spinal MRI showed a widened spinal canal and a distorted and atrophic spinal cord, due to the presence of multiple cysts with extensive leptomeningeal and dural contrast enhancement without the presence of solid masses (Figure 1B). Based on the history of large congenital melanocytic nevi (CMN) and the neuroradiological findings, a diagnosis of NCM was made.

**Figure 2:**
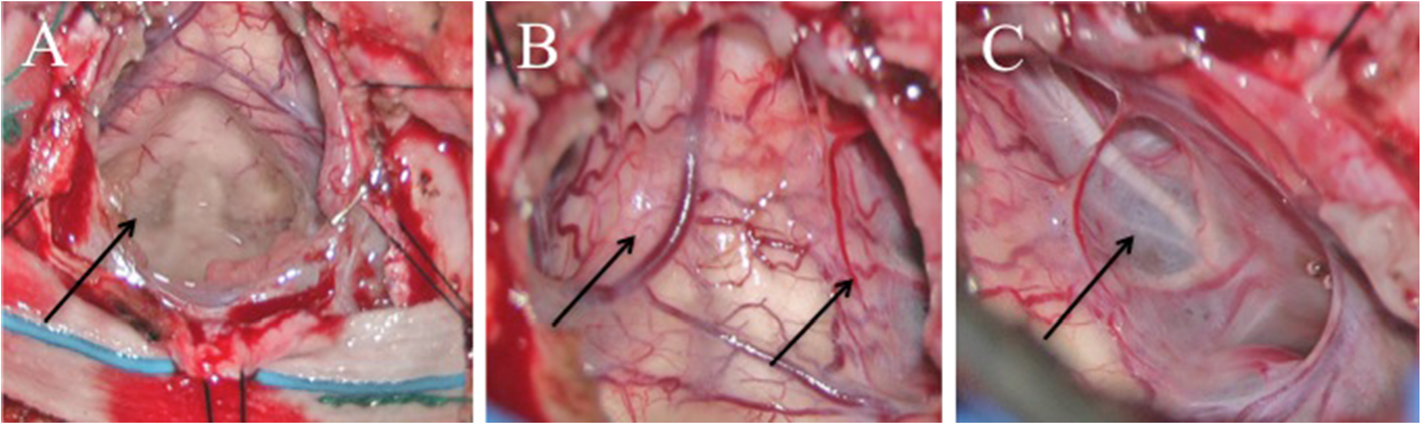
**Intra-operative photographs of the medulla spinalis and the spinal cord. A**. Intra-operatively, the floor of the fourth ventricle was covered with small cystic lesions (fourth ventricle, cervical medulla on top). **B**. Intra-operative photograph showing thickening and discoloration of the arachnoid around the spinal cord (image of part proximal of C1). **C**. Intra-operatively, the cyst wall covered all cranial nerves and arteries lateral to the brain stem, inhibiting normal flow of cerebrospinal fluid (accessory nerve and arteries lateral to the brain stem).

To decompress the brainstem and cerebellum and obtain material for histology, a suboccipital decompression was performed with drainage of the cerebellar cyst. Intra-operatively, the floor of the fourth ventricle was covered with small cystic lesions (Figure 2A), the arachnoid around the spinal cord was thickened and discolored (Figure 2B), and the cyst wall covered all cranial nerves and arteries lateral to the brain stem, inhibiting cerebrospinal fluid flow (Figure 2C).

Histopathological examination indeed showed leptomeningeal melanocytosis and thus confirmed the clinical diagnosis of NCM. Mutation analysis revealed a mutation in exon 3 at codon 61 of *NRAS* (c.1818C > A (p.(Gln61Lys) alias “Q61K”). On revision, the previously removed congenital nevi from both legs, left arm, right shoulder and right eyebrow were melanocytic nevi without signs of malignancy. *NRAS* mutation analysis of one of them showed an identical *NRAS* mutation as in the leptomeningeal melanocytosis. The *NRAS* mutation was absent in normal skin, thereby excluding an *NRAS* germ-line mutation. After surgery, the patient initially improved. In the following months, however, neurological symptoms increased and repeated imaging showed progressive leptomeningeal lesions around the brain and spinal cord with (further) compression of these structures and hydrocephalus (Figure 1C-D). In an attempt to relieve some of the symptoms, a ventriculo-peritoneal shunt was placed in the right lateral ventricle. However, the fourth ventricle enlarged again due to aquaduct stenosis. Hence, aquaduct stenting was performed. Unfortunately, this could not prevent further deterioration. At that time there were no neurosurgical options left and we refrained from radiotherapy since it was unlikely that irradiation would provide rapid clinical benefit.

In view of the previously described beneficial effect of MEK162 in *NRAS*-mutated metastatic melanoma, and knowing that our patient carried an activating *NRAS* mutation in his leptomeningeal melanocytosis lesion, we considered treatment of our patient with MEK162. Novartis agreed to supply MEK162 on compassionate use base, and after screening for eligibility and parental consent treatment with MEK162 45 mg BID was started.

In the following days however, the patient was readmitted to the hospital because of further deterioration with progressive general weakness and stridor. He had papules on the chest, consistent with mild skin toxicity due to MEK162. Cerebral MRI confirmed disease progression. Five days after starting MEK162 treatment, the boy died due to central neurogenic respiratory failure. The parents gave permission to perform autopsy.

#### Autopsy findings and histology

**Figure 3:**
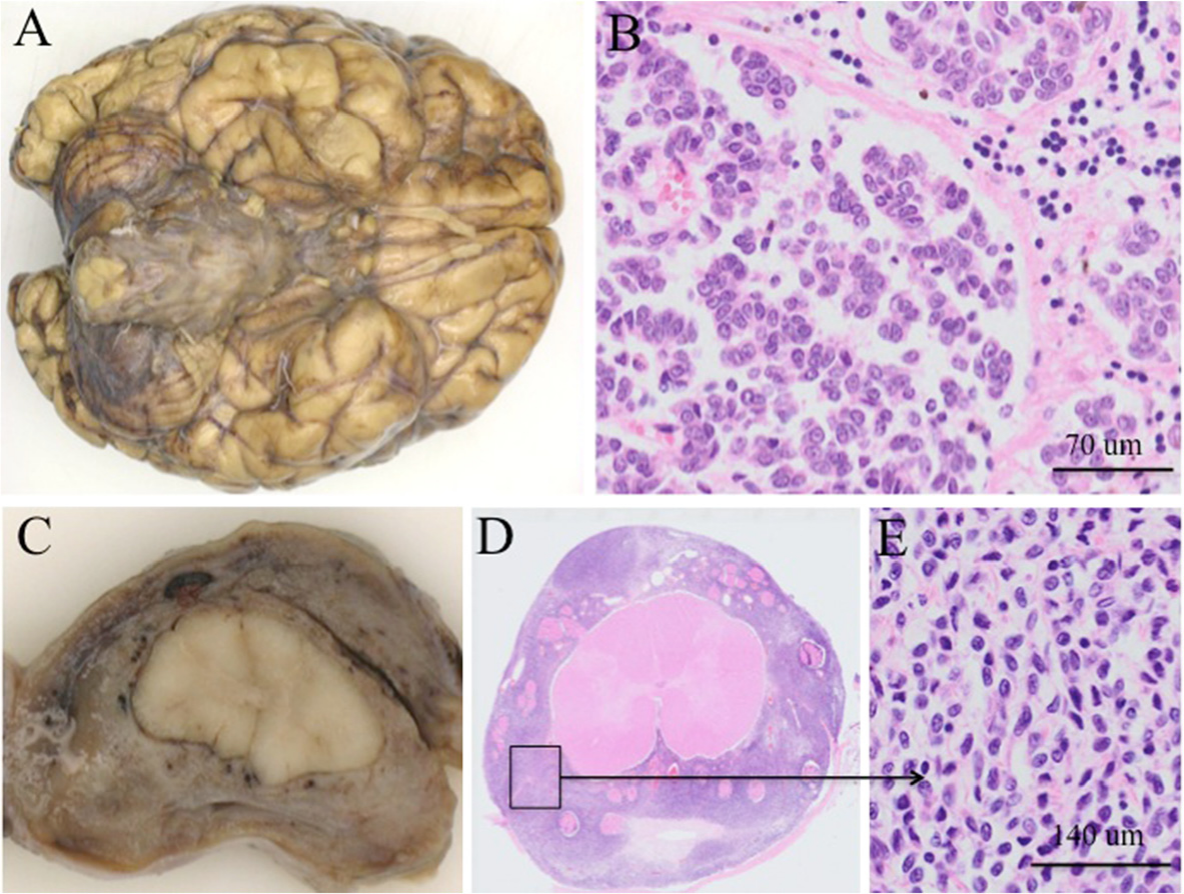
**Macro- and microscopy of the brain and the spinal cord obtained at autopsy. A**. The leptomeninges at the base of the brain were greyish and thickened, embedding blood vessels and cranial nerves. **B**. Microscopically, a leptomeningeal proliferation consisting of melanocytic cells was seen with extension of these cells in the perivascular (Virchow-Robin) spaces of the brain, without frank invasion of the CNS parenchyma. **C**. The spinal cord was surrounded by a thickened brown-greyish tumorous proliferation. This prominent leptomeningeal thickening extended along the entire spinal cord, resulting in a thick, plaque-like tumor compressing especially the cervical region of the spinal cord. **D**. Microscopy of a transverse section of the spinal cord. **E**. The melanocytic cells were ovoid and monotonous, with little cytonuclear atypia. Necrosis was absent and mitotic activity was low.

At autopsy, multiple large CMN were seen on the skin, mainly on the trunk, some with scars because of previous surgical removal. At the base of the brain (Figure 3A), around the cerebellar hemispheres and the brainstem, the leptomeninges were greyish and thickened, embedding blood vessels and cranial nerves, and obstructing the cerebellar cisterna and foramina. This prominent leptomeningeal thickening extended along the entire spinal cord resulting in a thick, plaque-like tumor compressing especially the cervical region of the spinal cord (Figure 3C). Some nodular thickening of leptomeninges around spinal nerve roots was present, while nerve roots from the cauda equina were completely embedded in leptomeningeal tumor. At cut surface of the brain, black discoloration was seen of the amygdala and focally in the right basal cortex.

Microscopically, a leptomeningeal proliferation consisting of melanocytic cells was seen (Figure 3B,D-E), confirmed by staining for S100, Melan-A and HMB-45. The melanocytic cells were ovoid and monotonous, with little cytonuclear atypia (Figure 3E) and occasionally some cytoplasmic melanin pigment. Necrosis was absent and mitotic activity was low. Extension of melanocytic cells in the perivascular Virchow Robin spaces of the brain (Figure 3B) and spinal cord was present, without frank invasion of the CNS parenchyma. Compression of the brain stem by the diffuse leptomeningeal melanocytosis, causing respiratory insufficiency by increased pressure, was considered as the cause of death. Histological examination of the nevi at autopsy and revision of previously removed CMN, demonstrated compound melanocytic nevi with congenital features, without signs of malignancy.

#### Immunohistochemistry

**Figure 4:**
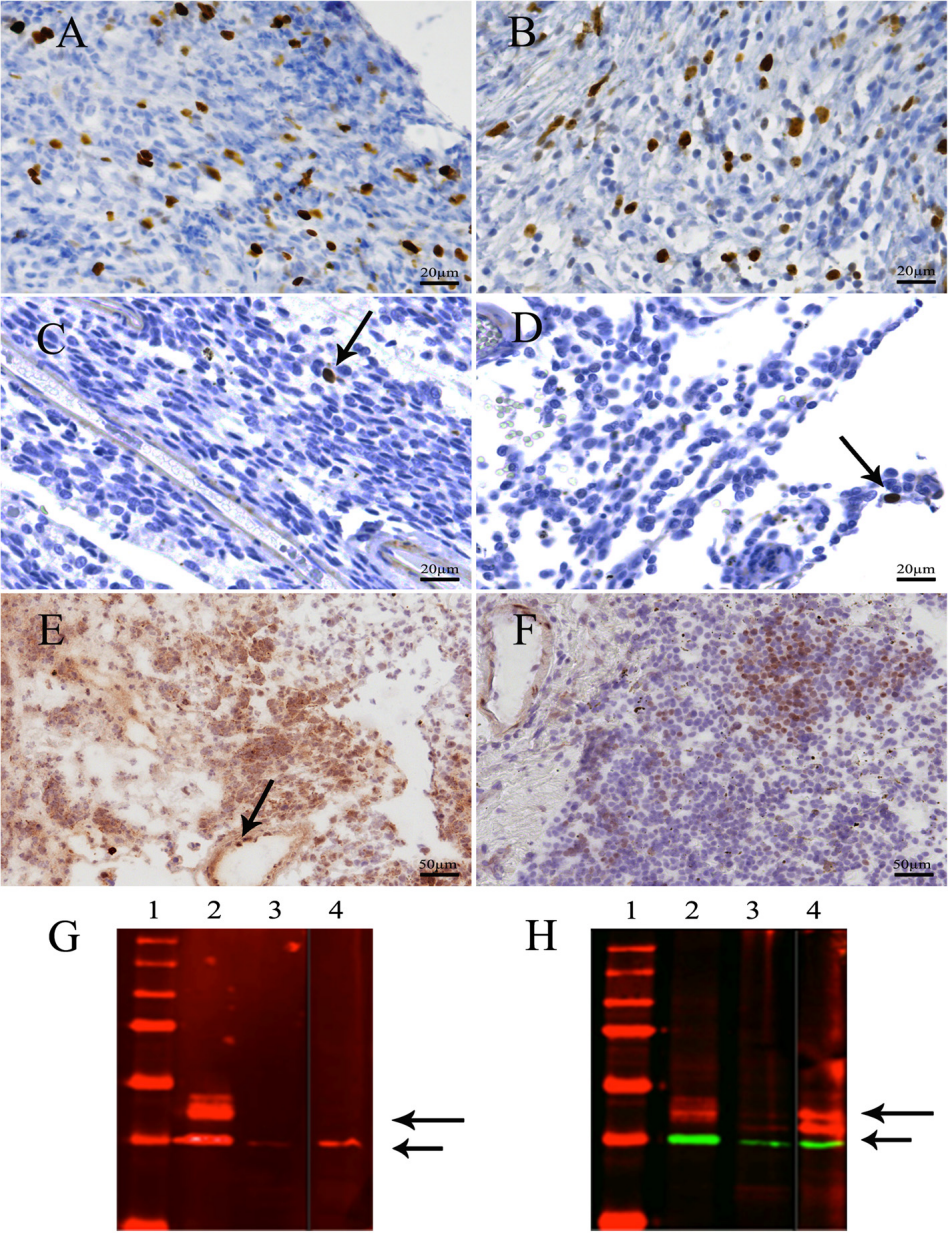
**Pre- and posttreatment MIB-1 and pERK stains and Western Blot analysis. A **and **B**. MIB-1 expression in the leptomeningeal melanocytosis sample before MEK162 treatment, in two hotspot regions (5% MIB-1-positive cells). **C** and **D**. The melanocytosis samples after MEK162 treatment showed lower MIB-1 expression than pre-treatment (<1% MIB-1-positive cells (arrows); compare with Figure 4**A** and **B**. **C** temporobasal brain and **D** frontobasal brain. **E**. pERK protein expression in the leptomeningeal melanocytosis before MEK162 treatment. Endothelial cells serve as a positive internal control (arrow). **F**. pERK protein expression after MEK162 treatment showing lower pERK expression than pre-treatment (compare with 4E). **G** and **H**. Western Blots showing expression of pERK **(G)** and total ERK **(H)** in the brain sample after MEK162 treatment (lane 4), along with Rh-18 and Granta-519 as positive and negative controls in lanes 2 and 3, respectively. In the brain sample, pERK cannot be detected after MEK162 treatment **(G)**, whereas total ERK is present **(H)**, as indicated by the long arrows. Short arrows indicate GAPDH expression.

To study whether MEK162 had sorted an effect on the leptomeningeal melanocytosis after 5 days of treatment, tumor tissue removed at operation before MEK162 treatment and tumor tissue at autopsy, was stained with the antibodies Ki67 (MIB-1) and pERK. Details on staining methods are presented in the Additional file 1. MIB-1 staining was performed on the pretreatment FFPE-sample and several postmortem FFPE-specimens of melanocytosis from different anatomic regions of the CNS (including cerebrum (parieto-occipital and frontobasal region), cerebellum, brainstem, different levels of spinal cord). The percentage of MIB-1-positive nuclei was semi-quantitatively assessed in hotspot regions at × 400 magnification counting at least 600 to 1000 cells. The pre-treatment sample contained several hotspots showing a MIB-1 Labelings Index (LI) of 5% (Figure 4A and B). In contrast, the melanocytosis samples after MEK162 treatment all showed lower MIB-1 expression with a MIB-1 LI of <1%, suggesting little tumor heterogeneity throughout the tumor as far as proliferative activity is concerned (Figure 4C and D).

Furthermore, 75% of tumor cells in the pre-treatment sample were positive in the pERK stain versus 25% of tumor cells in the post-treatment sample (Figure 4E, pre-treatment, and Figure 4F pERK staining post-treatment).

#### Mutation analysis

An identical mutation in exon 3 at codon 61 of *NRAS* (c.1818C > A (p.(Gln61Lys) alias “Q61K”) was present in the melanocytosis tissue removed at surgery and in one of the congenital nevi removed in the past, while normal skin did not reveal an *NRAS* mutation, thereby excluding an *NRAS* germ-line mutation. We also tested the melanocytosis sample for mutations in codon 600 of *BRAF* and for oncogenic mutations in codon 209 of the *GNAQ* and *GNA11* genes, as the latter are present in adult cases of primary leptomeningeal melanocytic neoplasms [8,9]. Mutations in these genes were absent.

#### Western Blotting

Unfortunately, the brain specimen before MEK162 treatment was insufficient for Western Blot analysis. In the brain sample that was obtained by autopsy the Western Blot showed presence of total ERK, but not of pERK, which is in line with the immunohistochemical staining results. See Figure 4G and 4H.

## Discussion

This case report describes a 13-year-old boy, known since birth with multiple large CMN, who developed progressive neurological symptoms due to leptomeningeal melanocytosis. The association of large and/or multiple CMN of the skin with melanocytic lesions of the CNS is known as NCM [1]. The CNS manifestations in NCM include leptomeningeal melanocytosis or melanoma, but also melanotic abnormalities with intraparenchymal deposits of melanin as well as associations with CNS malformations have been reported [1,10-12].

Leptomeningeal melanocytosis has a poor prognosis once neurological symptoms develop and treatment mostly includes surgical interventions to reduce intracranial pressure. The efficacy of irradiation and chemotherapy for this disorder is unclear [1].

Recently, it was demonstrated that early embryonic, post-zygotic somatic mutations in the *NRAS* gene are implicated in the development of NCM and childhood melanoma of the CNS [6,7,9]. Thus far, all primary childhood melanomas of the CNS and leptomeningeal melanocytosis were found to harbor somatic *NRAS* mutations: in the 6 cases reported in literature, including the present case, a codon 61 mutation in *NRAS* was demonstrated (1 case Q61L, 3 cases Q61R, 2 cases Q61K) [6,7,9]. It can be hypothesized that early during embryogenesis a melanocyte precursor acquires a somatic *NRAS* mutation resulting in a ‘mosaic’ pattern of melanocytes that colonize the skin and leptomeninges. This could explain the presence of an identical somatic *NRAS* mutation in the CMN as well as in the leptomeningeal melanocytosis in this patient.

Recently, a non-randomized phase-2 study has demonstrated that MEK162 shows activity in *NRAS*-mutated melanoma [13]. In addition, we previously reported on a mouse model in which embryonic induction of oncogenic *NRAS* mutation led to NCM-like disease in mice with development of primary CNS melanoma [6]. In that study, we were able to demonstrate that cells derived from the primary CNS melanomas in the mice displayed constitutive ERK activity and that those cells were sensitive to the MAP–ERK kinase (MEK) inhibitors PD184352, U0126, and AZD6244. Such information led us to start MEK162 treatment on compassionate use base in our patient. Unfortunately, the patient passed away only five days later and we were not able to assess the clinical effect of MEK162 treatment. However, immunohistochemical analysis revealed that after MEK162 treatment proliferation as well as pERK expression was lower in the leptomeningeal melanocytosis as compared to the pre-treatment situation. Of course these findings in a single patient should be interpreted with caution. Still, our findings are in line with those of another recent case report, reporting effect of vemurafenib in a patient with diffuse leptomeningeal melanoma metastasis carrying an activating *BRAF* mutation [14].

In the largest phase 2 study of MEK162 in melanoma patients so far, MEK162 was well tolerated overall [13]. The most common toxicities were skin- and gastrointestinal-related and fluid retention. These toxicities were generally mild and well manageable. More severe adverse events were uncommon; the most common grade 3–4 adverse event was an asymptomatic rise in creatine phosphokinase [13]. So far, no increased risk of paradoxical tumor development has been observed, as has been described with BRAF inhibitors [15]. Long-term data on safety of MEK162 are still awaited however.

In conclusion, we report a case of NCM with progressive symptomatic leptomeningeal melanocytosis harboring a somatic NRAS^Q61K^ mutation that was experimentally treated with MEK162. We observed a decrease in proliferation as well as a decrease in pERK expression in the post-treatment melanocytosis samples as compared to a pre-treatment sample, suggesting an effect of MEK inhibiting therapy. However, further studies are needed to determine whether MEK inhibitors can effectively target *NRAS*-mutated NCM. This is important considering the often fatal course of symptomatic NCM and the limited treatment options that are available for this disorder.

## Consent

Written informed consent was obtained from the mother of the patient for publication of this Case report and of accompanying images. A copy of the written consent is available for review by the Editor-in-Chief of Acta Neuropathologica Communications.

## Abbreviations

NCM: Neurocutaneous melanosis; CNS: Central nervous system; CMN: Congenital melanocytic nevus.

## Competing interests

The authors declare that they have no conflict of interests.

## Authors’ contributions

Each author has participated sufficiently in the work to take public responsibility for appropriate portions of the content. All authors read and approved the final version of the manuscript. HK and AW have substantially and equally contributed to conception and design of the report, to analysis and interpretation of data, and drafted and revised the manuscript. PG supervised the molecular tests and contributed to analysis and interpretation of these data as well as to writing the manuscript. BK, ML and PW contributed substantially to analysis and interpretation of data and results, and contributed to writing the manuscript. MD and JR performed the immunohistochemical stainings and genetic analyses and contributed to analysis and interpretation of these data. HD, MW and CH contributed to acquisition and interpretation of data and contributed to writing the manuscript. WB supervised the analyses, substantially contributed to conception and design of the report and to analysis and interpretation of data, and drafted and revised the manuscript.

## Supplementary Material

Additional file 1 Materials and methods including immunohistochemistry, Western Blot analysis and sequence analysis. Click here for file
