# Supplementary material for: Experimental treatment of NRAS-mutated neurocutaneous melanocytosis with MEK162, a MEK-inhibitor
Source: Acta Neuropathol Commun. 2014 Apr 8;2:41. doi: 10.1186/2051-5960-2-41 (PMC4023633; doi:10.1186/2051-5960-2-41)
Supplement: Additional file 1 — Materials and methods including immunohistochemistry, Western Blot analysis and sequence analysis. [file 2051-5960-2-41-S1.doc]

**Materials and methods**

***Histology and immunohistochemistry***

Material of the tumor at operation and autopsy was formalin-fixed and paraffin-embedded (FFPE) and partially snap frozen.

The FFPE-tumor was stained with H&E, Ki-67 (DAKO-M7240, clone MIB-1; dil. 1:100; with antigen retrieval, citrate 10’), MART (Immunologic, ILM022111-C1; dil.1: 100; with antigen retrieval, EDTA 10’), and HMB-45 (Leica-Menarini, NCL-L-HMB45; dil.1: 20; with antigen retrieval, citrate 10’). Frozen material of the leptomeningeal melanocytosis from the operation and at autopsy was stained with pERK (Cell Signalling, clone #4376; dil. 1:100). The latter detects endogenous levels of p44 and p42 ERK1 and ERK2 when dually phosphorylated at Thr202 and Tyr204 of Erk1 (and Thr185 and Tyr187 of Erk2), and singly phosphorylated at Thr202.

***Tumor DNA extraction and sequence analysis***

All procedures involving the use of human tissue were in accordance with valid standards for this type of investigation in The Netherlands. Three manually dissected sections of 10μm FFPE tissue with an estimated tumor cell percentage of at least 50% were used for DNA extraction. DNA extraction and sequence analysis of *NRAS* were conducted as previously described. All PCR reactions were conducted in duplicate using 2 independent PCR products for sequence analysis.

***Protein lysates and Western blotting*** Protein lysates of the brain sample after MEK162 treatment and of the positive and negative cell line controls were prepared in RIPA Buffer (#9806s, Cell Signaling, Leiden Netherlands). The Rh-18 cell line was used as a positive control (human rhabdomyosarcoma cell line) and the Granta-519 cell line as a negative control (mantle cell lymphoma cell line). Of the protein lysates 25 µg was loaded on a 10% polyacrylamide gel. Gel electrophoresis and Western blotting onto a nitrocellulose Hybond-C extra membrane (RPN303E, GE Healthcare Europe GmbH, Diegem Belgium) was performed according to standard procedures. After transfer of the proteins, the membrane was blocked using blocking buffer (927-40000, LICOR, Lincoln USA) for 1 hr. at room temperature (rt), and incubated with phospho-p44/42 MAPK (Erk1/2) mouse monoclonal antibody E10 as primary antibody (#9106; Cell Signalling, Danvers, MA, USA), overnight at 4°C, washed with Tris-buffered saline (TBS), subsequently incubated with a Alexa Fluor goat-anti-mouse 680 antibody (A21057, Molecular Probes, Bleiswijk, the Netherlands) for 1 hr. at rt and washed with TBS again. A similar blot was made using the total p44/42 MAPK (Erk1/2) rabbit polyclonal antibody (#9102; Cell Signalling, Danvers, MA, USA) as primary antibody. The blots were scanned using the Odyssey instrument (ODYSSEY CLx infrared imaging system, LICOR, Lincoln USA). After scanning, the blot was incubated with GAPDH mouse monoclonal antibody to assess protein loading (clone 6C5, # AB8245, Abcam, Cambridge, UK) for 1 hr. at rt, washed with TBS, and further incubated with goat anti-mouse 680 antibody (blot A) or goat-anti-mouse 800 antibody (#35521, Thermoscientific, Waltham, MA, USA; blot B), washed and scanned using the Odyssey.

**References**

1. Casparie M, Tiebosch AT, Burger G, Blauwgeers H, van de Pol A, van Krieken JH, Meijer GA: **Pathology databanking and biobanking in The Netherlands, a central role for PALGA, the nationwide histopathology and cytopathology data network and archive Cellular oncology.** *Cell Oncol* 2007, **29**:19-24.

2. Kusters-Vandevelde HV, Klaasen A, Kusters B, Groenen PJ, van Engen-van Grunsven IA, van Dijk MR, Reifenberger G, Wesseling P, Blokx WA: **Activating mutations of the GNAQ gene: a frequent event in primary melanocytic neoplasms of the central nervous system.** *Acta Neuropathol* 2009, **119:** 317-23.
